# Papillary renal neoplasm with reverse polarity and renal clear cell carcinoma: a case report of image–pathology–molecular multidimensional diagnosis and nephron sparing surgery practice

**DOI:** 10.3389/fonc.2025.1739206

**Published:** 2026-01-16

**Authors:** Yuxuan Liu, Yanchen Wang, Lihui Guan, Shuping Sun, Huimin Sun, Yaofei Sun

**Affiliations:** 1 School of Clinical Medicine, Shandong Second Medical University, Weifang, China; 2 Department of Urology, Weifang People’s Hospital, Weifang, Shandong, China; 3 Shandong Provincial Key Laboratory for Prevention and Treatment of Urological Diseases in Medicine and Health, Weifang, Shandong, China; 4 Oncology Department, Laixi People’s Hospital, Tsingtao, Shandong, China; 5 Precision Pathology Diagnosis Center, Weifang People’s Hospital, Weifang, Shandong, China

**Keywords:** GATA3, ipsilateral double primary renal cell carcinoma, papillary renal neoplasm with reverse polarity (PRNRP), RCCC, renal clear cell carcinoma

## Abstract

Papillary renal neoplasm with reverse polarity (PRNRP) is a new pathological type of renal tumor, and its incidence is low. It is considered to be an indolent renal tumor at present. It is rare for PRNRP to co-occur with other tumors in the same kidney. Like other renal tumors, patients with PRNRP do not have any specific clinical manifestations. During surgery, we resected a solid and a cystic lesion in the left kidney simultaneously, and performed two partial nephrectomy on the ipsilateral kidney at one time, both with good surgical results. The postoperative pathological results were as follows: (1) solid lesions are PRNRP, and (2) cystic lesions are RCCC. Here, we present a case of PRNRP concurrent with RCCC and review the literature.

## Introduction

In the 2022 WHO new classification, papillary renal cell carcinoma (pRCC) is not recommended to be classified into type 1 and type 2 subtypes. In addition to typical pRCC, there are some renal cell carcinomas (RCCs) with special molecular mutations or special pathological structures, such as MiT family translocation RCC or fumarate hydratase (FH)-deficient RCC (1, 2). In 2019, a team proposed a new type of papillary renal neoplasm with reverse polarity (PRNRP) based on its special nuclear manifestations and gene expression (3). This is a novel pathological type of renal tumor, which mainly shows a renal tubular pattern, consisting of a single layer of cuboidal epithelial cells lined by a monolayer of eosinophils, and a round nucleus appearing at the tip. Immunohistochemically, this tumor shows L1CAM expression and has a recurrent KRAS mutation (4, 5). It has been suggested that the tumor may originate from the distal renal tubules, especially the cortical collecting ducts, and may retain its cell polarity, except for nuclear inversion (6). It has been confirmed that the pathological features of PRNRP are like those of tubular structures with frequent cystic changes, low-grade tumor cells with eosinophilic cytoplasm and inverted nuclear location, and diffuse and strong expression of GATA3 and 34βE12 (7–9). The low proliferation index of Ki-67 indicates a good prognosis (10). However, Vimentin and CD10 were negative (11, 12). Other articles have shown that its tumors may also have a performance similar to that of clear cell carcinoma, but they rarely show psammoma bodies, necrosis, mitotic figures, and intracellular hemosiderin (13).

In clinical diagnosis, we still need to rely on imaging examination, mainly computed tomography (CT) and magnetic resonance imaging (MRI). CT findings were as follows: pseudocapsule, CT plain scan turned into isodensity or slightly hyperdensity shadow. MRI showed low signal on T2WI and mild diffusion limitation on DWI (diffusion-weighted imaging). All cases showed mild or moderate enhancement in the arterial phase, and then further progressive enhancement in the venous phase and delayed phase (14).

Clinically, positive GATA3 staining and KRAS mutation remain the main indicators for the diagnosis of PRNRP, which are found in more than 80% of reported cases. Because of its satisfactory prognosis, monitoring and follow-up of patients with PRNRP should be additionally formulated (3, 4).

Renal clear cell carcinoma (RCCC) is the most common pathological type of RCC. The characteristic gene of RCCC is von Hippel–Lindau (VHL) tumor suppressor gene inactivation (15–17). RCCC accounts for approximately 5% of all malignant tumors, and is the 6th most common malignant tumor in men and the 10th most common malignant tumor in women (18). According to the 2020 Global Cancer statistics, the incidence and mortality of kidney cancer were 431,288 and 179,368 cases, respectively. RCC originates from renal tubular epithelial cells and accounts for more than 90% of renal cancers (19). Most of the disease has no obvious clinical symptoms and is detected by imaging examination. Clinically, 70% of the patients are in stage I, and 11% are in stage IV (15). Most patients with early-stage tumors can achieve good treatment results after receiving surgical treatment (20). For patients with tumors less than 4 cm (cT1a), the 5-year survival rate after partial nephrectomy can reach 94% (15).

There is no clear explanation for the introduction and specific treatment of ipsilateral double primary RCC with different pathological types. The authors believe that the two lesions should be treated at the same operation on the premise of ensuring the safety of patients.

## Case introduction

**Figure 1:**
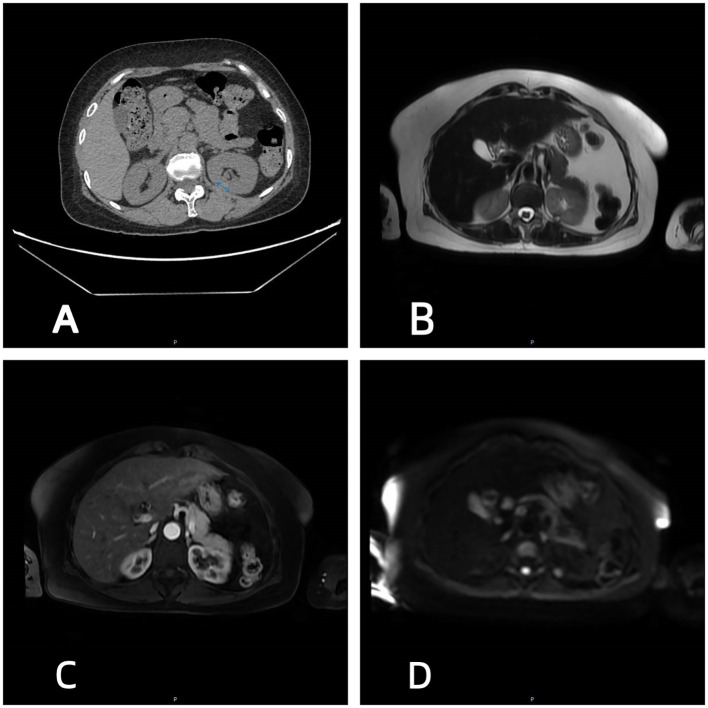
CT and MRI imaging of PRNRP.

**Figure 2:**
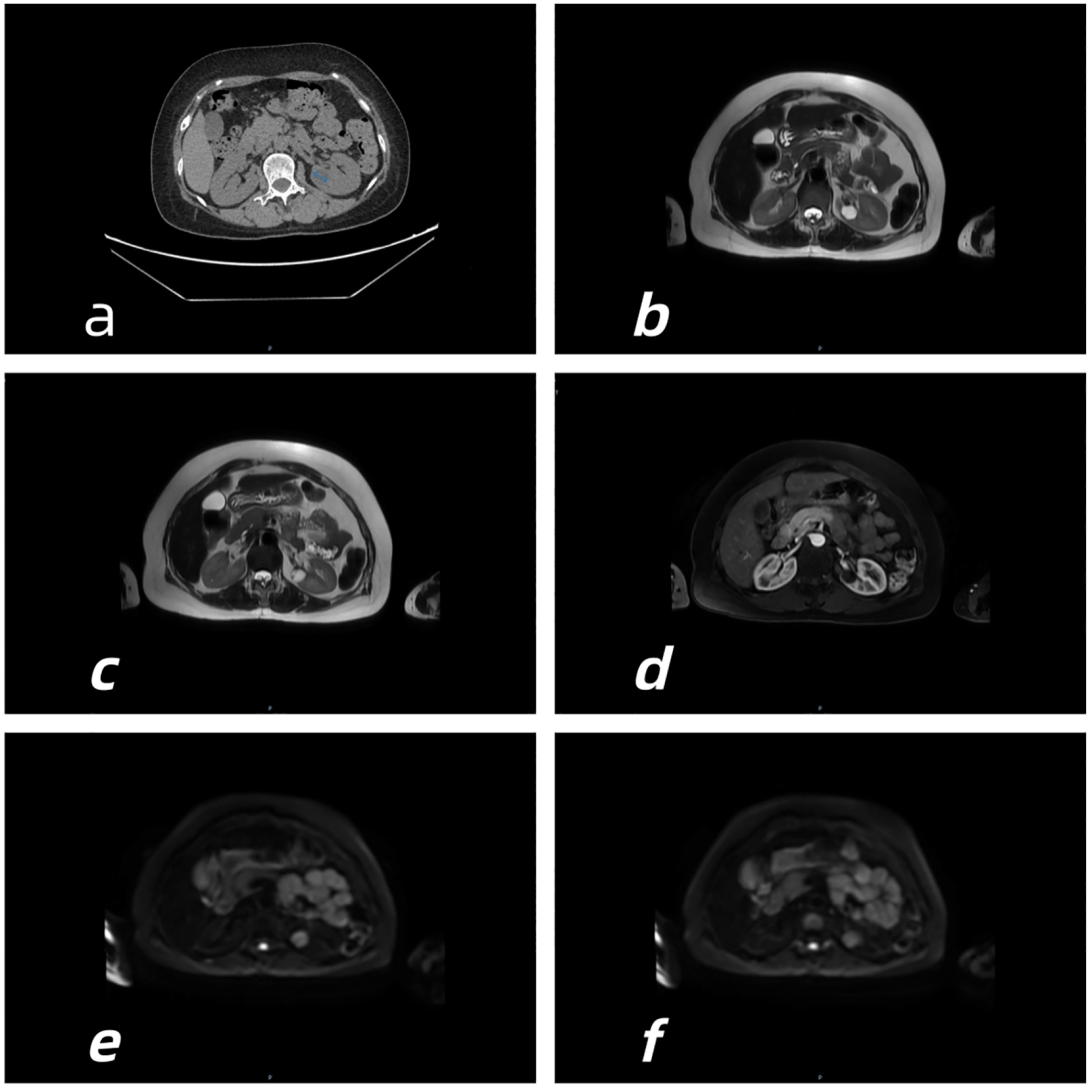
CT and MRI imaging of RCCC.

A 55-year-old female farmer was admitted to the Department of Respiratory Medicine of our hospital due to fever and cough. Abdominal CT scan was performed to incidentally find a left renal mass. One month later, the patient visited the urology department of our hospital again, and the relevant imaging examination was performed in our department. CT showed that there was a first density shadow on the lumbar side of the left kidney (**Figure 1A**), and there was a slightly lower density shadow on the lumbar kidney lip below it (**Figure 2A**). MRI showed that there was a T2WI low signal lesion in the lumbar side of the left kidney, and the enhancement was not obvious in the arterial phase. DWI showed a slightly hyperintense signal (**Figures 1B–D**). Below it, there was a well-defined hyperintense T2WI shadow, without obvious enhancement in the arterial phase. DWI showed hyperintensity (**Figures 2B–F**). Laparoscopy-assisted partial nephrectomy was performed under general anesthesia with endotracheal intubation on 21 April 2025. During the operation, partial nephrectomy was performed on both lesions at one time, and the specimens were immediately sent to the pathology department. The operation was successful and the patient returned to the ward safely after operation.

**Figure 3:**
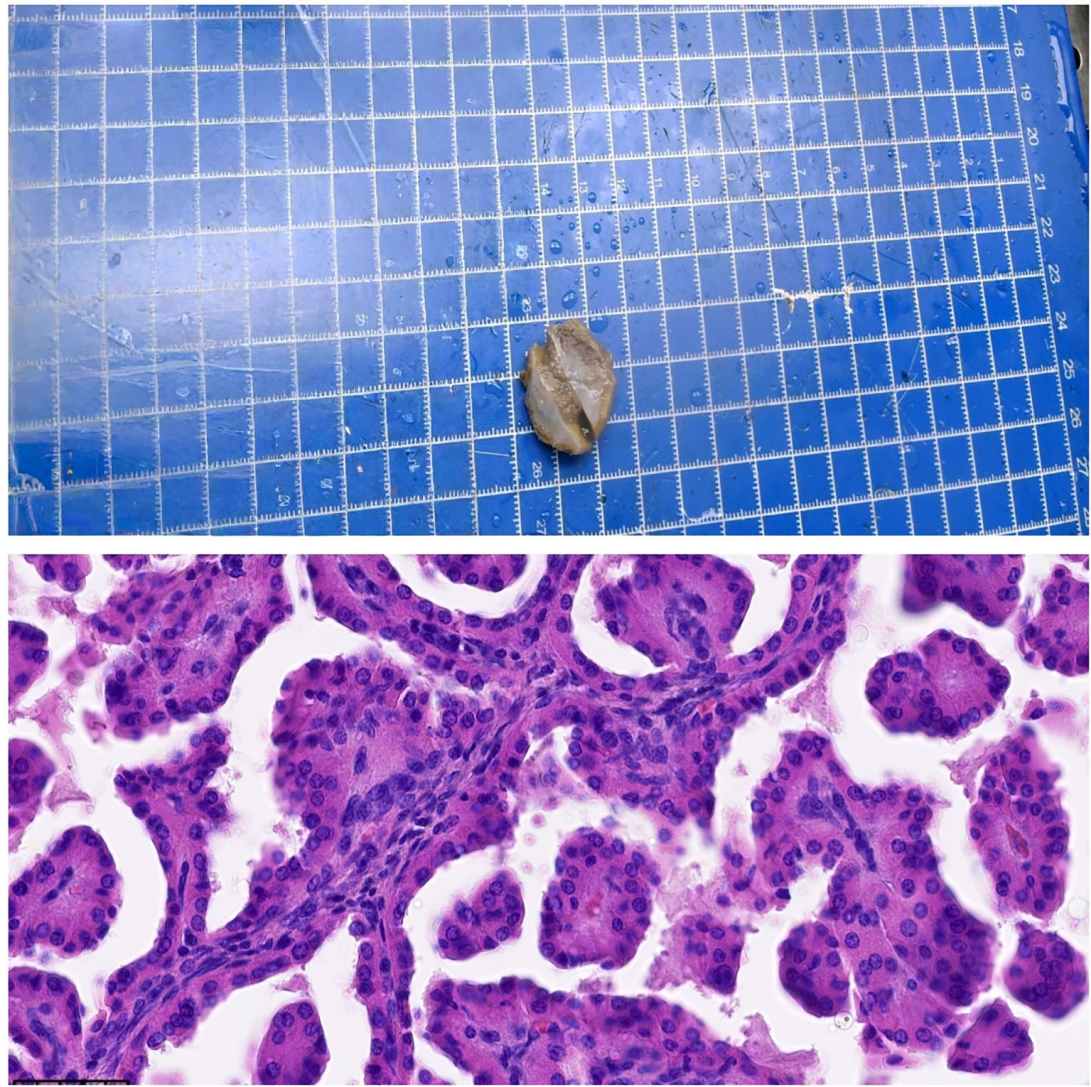
The pathological manifestations of PRNRP.

**Figure 4:**
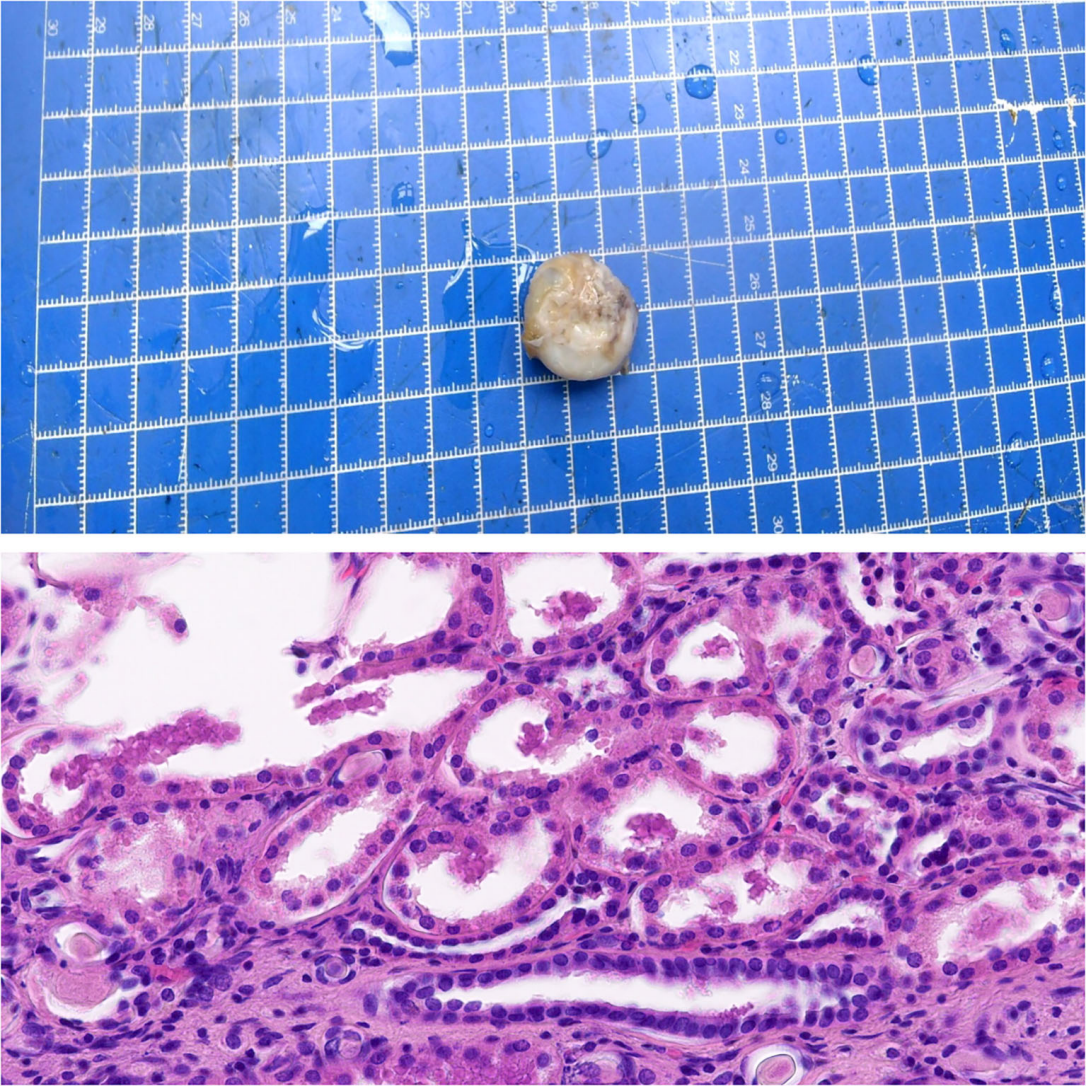
The pathological manifestations of RCCC.

Six days later, the pathological results were as follows: (left renal solid mass) size 1.5 cm × 1.2 cm × 0.8 cm (**Figure 3**), WHO/ISUP (World Health Organization/International Society of Urological Pathology) grade I; combined with histological morphology and immunohistochemistry, we considered that papillary renal tumor with nuclear polarity inversion (PRNRP) could not be excluded, and KRAS gene detection was needed for further diagnosis. There was no accumulation of renal capsule, vascular tumor thrombus, and nerve invasion. The peripheral and basal margins of the tumor were negative. Immunohistochemical study: PAX-8 (+), CK7 (+), P504S (+), FH (+), SDHB (+), GATA3 (+), E-Cadherin (+), EMA (+), CAIX (−), CD10 (partially +), CD117 (partially +), Vimentin (−), and Ki-67 (index 5%) (**Figure 3**). Left renal cystic mass was consistent with cystic RCCC combined with immunohistochemistry. The tumor size was 2.2 cm × 2 cm × 1.2 cm (**Figure 4**), WHO/ISUP(World Health Organization/International Society of Urological Pathology) grade I, and there was no vascular tumor thrombus and nerve invasion. Immunohistochemical study showed CAIX (−), PAX-8 (+), CD10 (partially +), CK7 (+), and GATA3 (−) (**Figure 4**).

The patient basically recovered and was discharged from hospital 6 days after surgery. The incision recovered well at discharge, and no adverse consequences occurred during follow-up. The patient was asked to be reexamined in the hospital 3 months later.

## Discussion

As a rare and new type of renal cell tumor, PRNRP has only been described in very few cases (3, 21), and the current clinical common PRNRP is a small, multi-encapsulated, cystic tumor (22), which is more common in middle-aged and elderly people (46–84 years old) without obvious gender preference. It rarely occurs in two kidneys at the same time, and it is rarely concurrent with other tumors. At present, it is mostly found accidentally during physical examination, because most patients have no obvious symptoms (6, 11, 13, 23). Most of them are 0.8–8 cm in diameter and have clear boundaries with the pseudocapsule (13, 22). Four diagnostic criteria for PRNRP have been proposed (1): thin or tubular growth with predominant protrusion (2); focal or diffuse interstitial vitrification (3); granular cytoplasm of eosinophils; and (4) the tumor nuclei are neatly arranged on the top of the cytoplasm away from the basement membrane, showing the characteristics of “reverse polarity”, the same size, and low nuclear grade (23).

According to the article, KRAS mutation is strongly correlated with the occurrence of PRNRP and is an early event in the development of this tumor (24). Literature review showed that 78 of 92 PRNRP cases (84.8%) carried KRAS mutation, which is the only known renal cell tumor with KRAS mutation (6, 8, 9, 12, 23–26).

Based on the literature review and the study of this case, the author believes that PRNRP should be considered as a new pathological type of RCC combined with histological and other related analysis, and GATA3 should be used as a routine pathological screening test for RCC. Although PRNRP is sometimes difficult to distinguish from other pRCC morphologically, it is necessary to make GATA3 a new pathological type of RCC. However, pRCC generally does not have KRAS mutation (27), so KRAS gene detection can be used to distinguish PRNRP from other PRCCs when necessary.

So far, no consensus has been reached and no clear steps have been proposed for the treatment of PRNRP. Surgical resection is still the preferred treatment for any non-metastatic solid renal tumor, and minimally invasive surgical resection should be performed if conditions are available (28). The choice of surgical methods—radical nephrectomy (RN) and partial nephrectomy (PN)—are the main choices. For cT1 and some cT2 with a low renal score, partial nephrectomy can be selected under the premise of ensuring negative surgical margins. Compared with radical nephrectomy, partial nephrectomy can not only completely remove the tumor, but also preserve as much normal nephron as possible, reduce the incidence of long-term renal insufficiency, and even reduce the incidence of cardiovascular events, and greatly improve the quality of life of patients after surgery (29–32). All patients undergoing this surgery should undergo pathological biopsy to confirm the precise pathological diagnosis of renal tumors and effectively guide postoperative rehabilitation and follow-up.

For RCCC, early partial nephrectomy or timely radical nephrectomy has become a consensus in the industry. Standard management for RCCC is well-established; thus, the author believes that there is no need to discuss the surgical treatment of RCCC.

In this case, partial nephrectomy was performed for both primary tumors on the same side of the kidney. At the same time, the “triple win” of PN was ensured, and good surgical results were achieved. Even if another cystic lesion was reported as RCCC in postoperative pathology, it was still proved that it was clinically feasible to perform two lesion resections after one renal artery clamping. However, because of the small number of cases of this type and the lack of large-scale clinical testing, it is still uncertain whether divided operation and single operation have significant adverse effects on long-term prognosis. The author will continue to follow up this patient after surgery to evaluate the long-term effect of surgery.

## Conclusions

Although PRNRP is a new pathological type, early partial nephrectomy is still considered to be the first choice for its treatment. As with RCCC, early intervention can achieve good therapeutic effects. This case also proved that the two primary lesions on the same side can be completely removed at the same time in a single operation with good results.

## Data Availability

The original contributions presented in the study are included in the article/supplementary material. Further inquiries can be directed to the corresponding author.
